# Supplementary material for: ICP-MS Multi-Elemental Analysis of the Human Meninges Collected from Sudden Death Victims in South-Eastern Poland
Source: Molecules. 2022 Mar 15;27(6):1911. doi: 10.3390/molecules27061911 (PMC8949131; doi:10.3390/molecules27061911)
Supplement: Supplementary file 1 [file molecules-27-01911-s001.zip › molecules-1611244-supplementary.pdf]

Calibration for D:\ICPMH\1\DATA\mozg_24-05-2021.b\001CALB.d

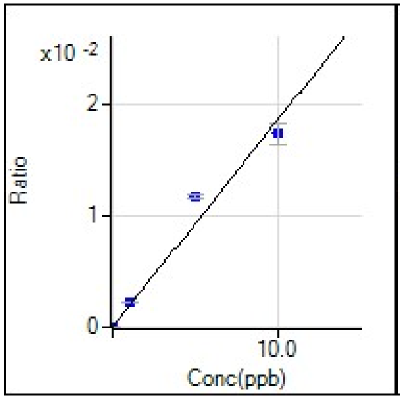

9 Be [No Gas]
ISTD: 45 Sc
y = 1.858E-3 x + 2.144E-6
R 0.9836
DL 0.0007641
BEC 0.001154

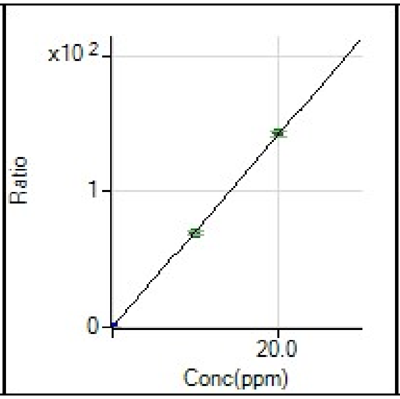

23 Na [He]
ISTD: 45 Sc
y = 7.074E0 x + 7.326E-2
R 0.9999
DL 0.001204
BEC 0.01036

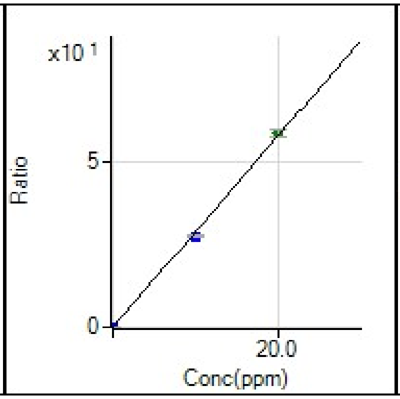

24 Mg [He]
ISTD: 45 Sc
y = 2.894E0 x + 8.677E-4
R 0.9995
DL 0.0004162
BEC 0.0002998

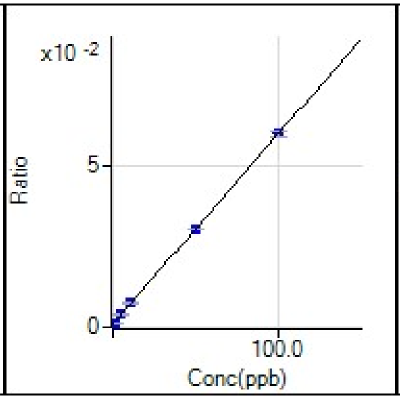

27 Al [He]
ISTD: 45 Sc
y = 5.877E-4 x + 1.065E-3
R 0.9999
DL 1.114
BEC 1.812

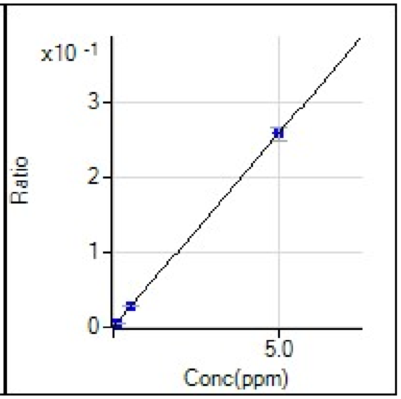

31 P [He]
ISTD: 45 Sc
y = 5.159E-2 x + 5.813E-4
R 1.0000
DL 0.01195
BEC 0.01127

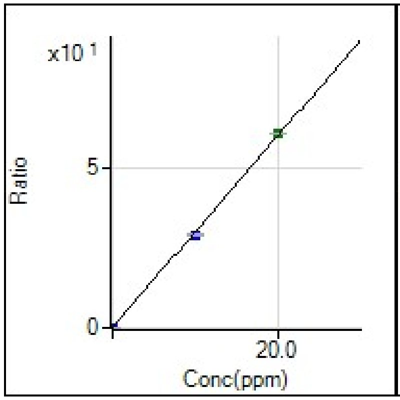

39 K [He]
ISTD: 45 Sc
y = 3.002E0 x + 1.911E-1
R 0.9997
DL 0.004805
BEC 0.06365

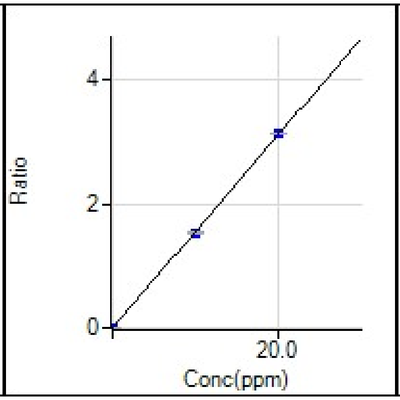

44 Ca [He]
ISTD: 45 Sc
y = 1.560E-1 x + 2.042E-3
R 0.9999
DL 0.008008
BEC 0.01309

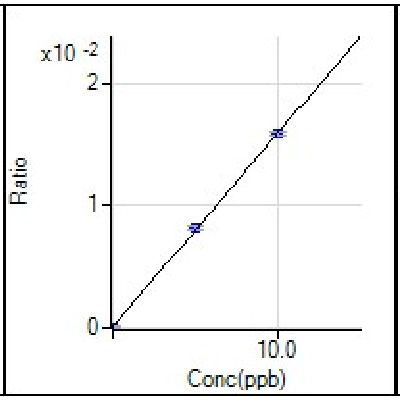

47 Ti [He]
ISTD: 45 Sc
y = 1.581E-3 x + 7.456E-5
R 0.9999
DL 0.04165
BEC 0.04715

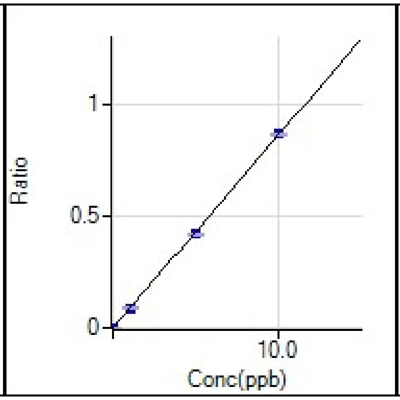

51 V [He]
ISTD: 45 Sc
y = 8.614E-2 x + 1.915E-4
R 0.9999
DL 0.004488
BEC 0.002223

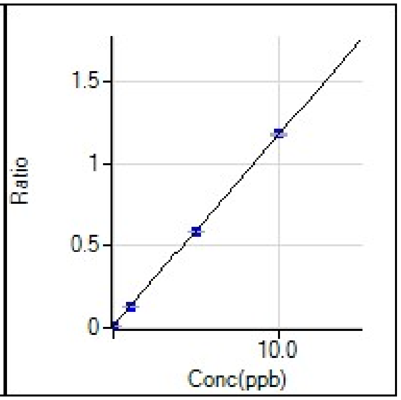

52 Cr [He]
ISTD: 45 Sc
y = 1.163E-1 x + 1.454E-2
R 1.0000
DL 0.03845
BEC 0.125

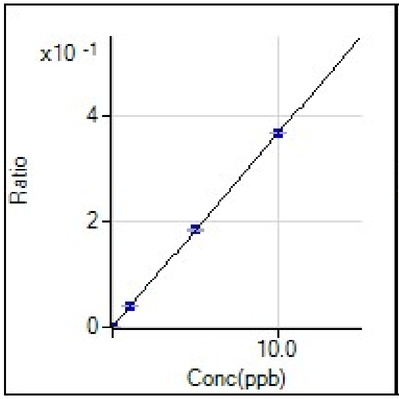

55 Mn [He]
ISTD: 45 Sc
y = 3.652E-2 x + 4.934E-4
R 1.0000
DL 0.02262
BEC 0.01351

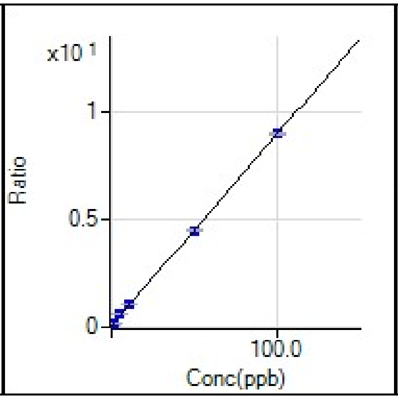

56 Fe [He]
ISTD: 45 Sc
y = 8.925E-2 x + 8.309E-2
R 0.9999
DL 1.429
BEC 0.9309

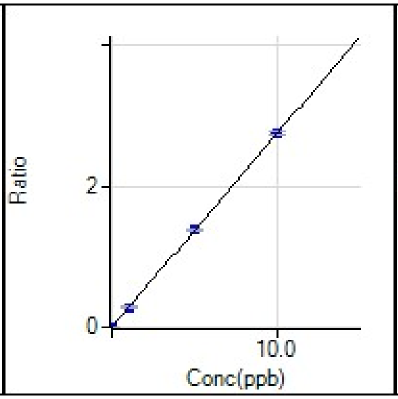

59 Co [He]
ISTD: 45 Sc
y = 2.748E-1 x + 1.654E-4
R 1.0000
DL 0.001149
BEC 0.0006018

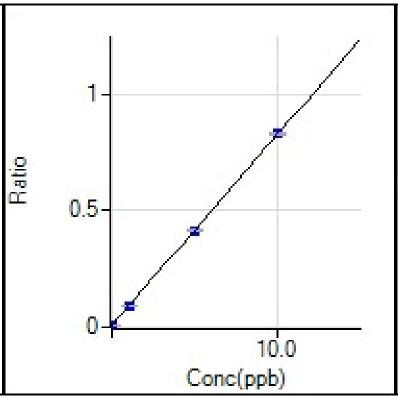

60 Ni [He]
ISTD: 45 Sc
y = 8.195E-2 x + 8.825E-3
R 1.0000
DL 0.02481
BEC 0.1077

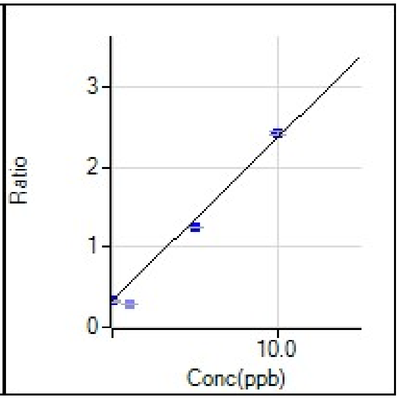

63 Cu [He]
ISTD: 45 Sc
y = 2.029E-1 x + 3.316E-1
R 0.9975
DL 0.2775
BEC 1.634

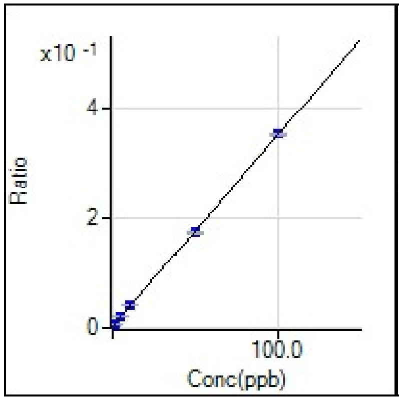

66 Zn [He]
ISTD: 89 Y
y = 3.474E-3 x + 3.073E-3
R 0.9999
DL 0.1832
BEC 0.8845

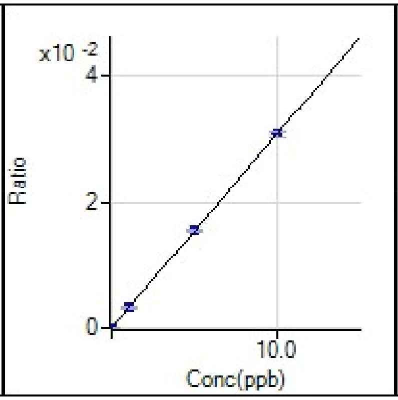

71 Ga [He]
ISTD: 89 Y
y = 3.078E-3 x + 1.292E-5
R 1.0000
DL 0.01327
BEC 0.004196

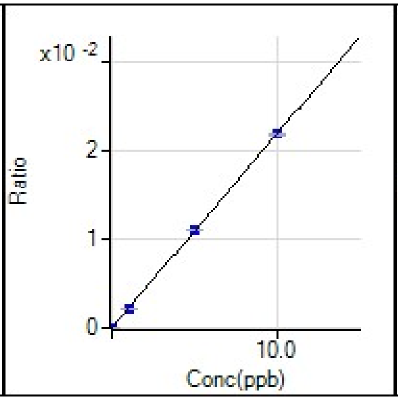

75 As [He]
ISTD: 89 Y
y = 2.199E-3 x + 5.150E-6
R 1.0000
DL 0.005237
BEC 0.002341

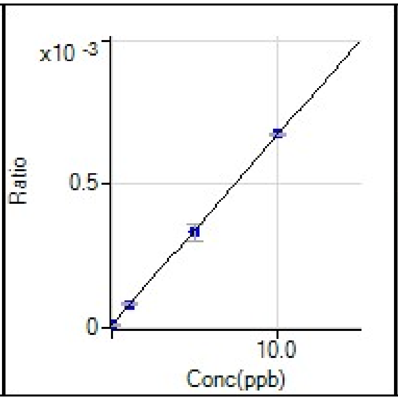

78 Se [He]
ISTD: 89 Y
y = 6.587E-5 x + 1.184E-5
R 0.9998
DL 0.27
BEC 0.1797

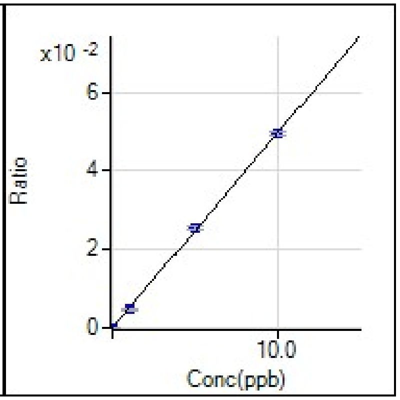

85 Rb [He]
ISTD: 89 Y
y = 4.965E-3 x + 3.350E-5
R 0.9998
DL 0.01285
BEC 0.006749

Calibration for D:\ICPMH\1\DATA\mozg_24-05-2021.b\001CALB.d

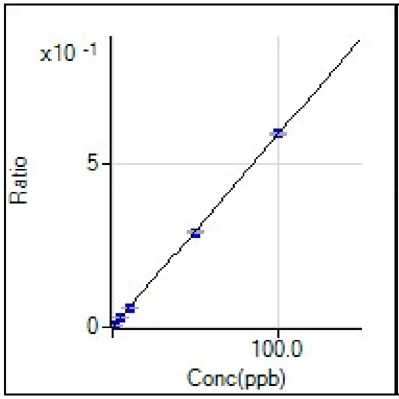

88 Sr [He]
ISTD: 89 Y
y = 5.884E-3 x + 1.200E-3
R 1.0000
DL 0.08089
BEC 0.2039

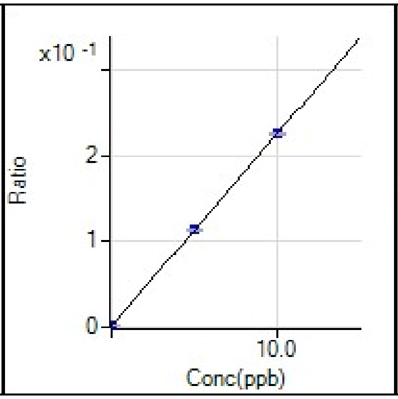

90 Zr [He]
ISTD: 89 Y
y = 2.244E-2 x + 1.519E-3
R 1.0000
DL 0.02611
BEC 0.06767

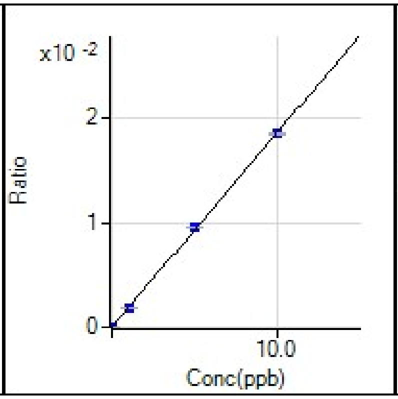

75 -> 91 As [O2]
ISTD: 89 -> 105 Y
y = 1.859E-3 x + 1.175E-5
R 0.9999
DL 0.001647
BEC 0.006319

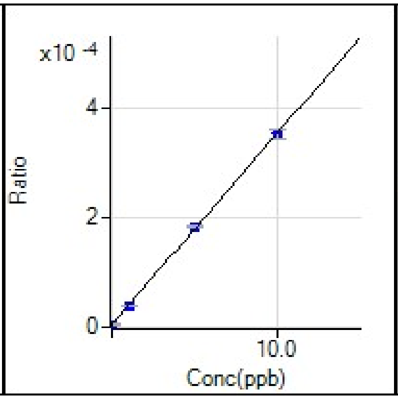

78 -> 94 Se [O2]
ISTD: 89 -> 105 Y
y = 3.498E-5 x + 5.170E-6
R 0.9999
DL 0.09113
BEC 0.1478

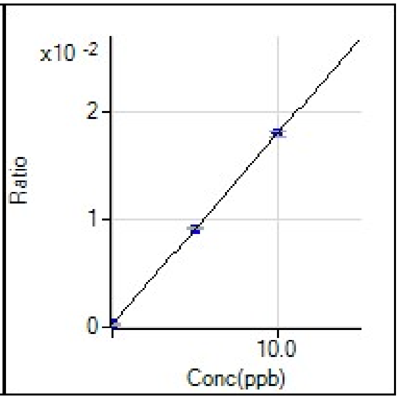

95 Mo [He]
ISTD: 89 Y
y = 1.775E-3 x + 2.754E-4
R 1.0000
DL 0.1721
BEC 0.1551

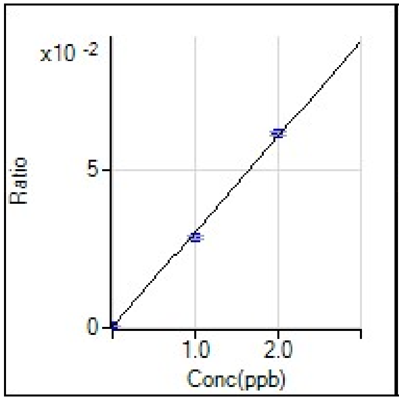

105 Pd [He]
ISTD: 89 Y
y = 3.011E-2 x + 4.265E-4
R 0.9993
DL 0.005865
BEC 0.01416

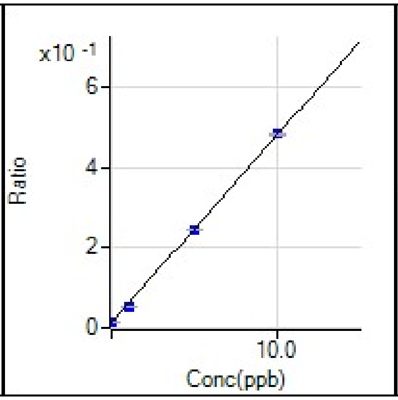

107 Ag [He]
ISTD: 89 Y
y = 4.683E-2 x + 1.404E-2
R 0.9998
DL 0.03558
BEC 0.2997

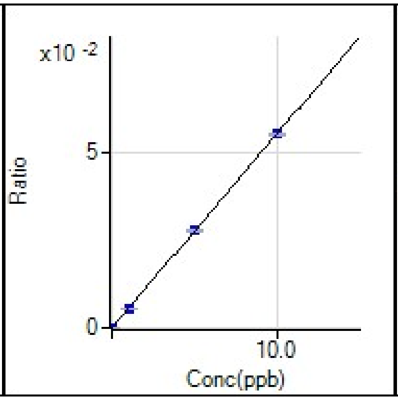

111 Cd [He]
ISTD: 89 Y
y = 5.554E-3 x + 3.085E-6
R 1.0000
DL 0.001278
BEC 0.0005554

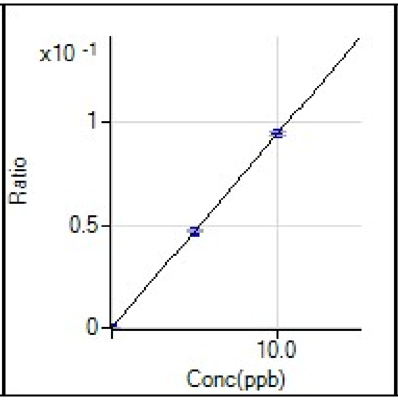

118 Sn [He]
ISTD: 89 Y
y = 9.408E-3 x + 1.210E-4
R 1.0000
DL 0.00713
BEC 0.01287

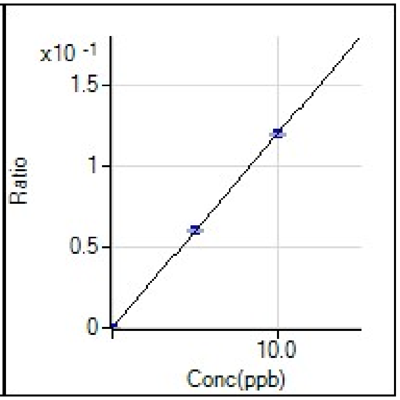

121 Sb [He]
ISTD: 89 Y
y = 1.196E-2 x + 3.166E-5
R 1.0000
DL 0.006776
BEC 0.002648

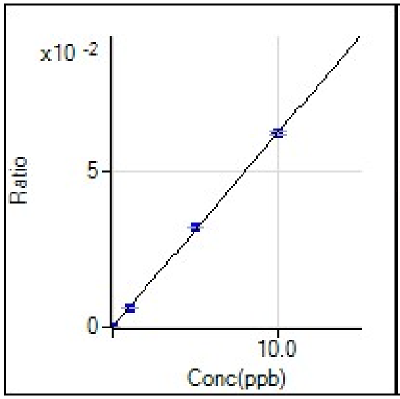

133 Cs [He]
ISTD: 175 Lu
y = 6.266E-3 x + 1.282E-5
R 0.9999
DL 0.005378
BEC 0.002046

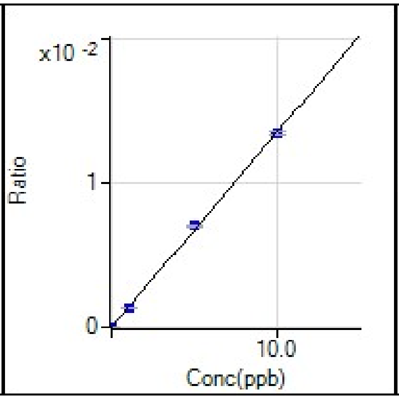

137 Ba [He]
ISTD: 175 Lu
y = 1.349E-3 x + 8.743E-6
R 0.9997
DL 0.01266
BEC 0.006482

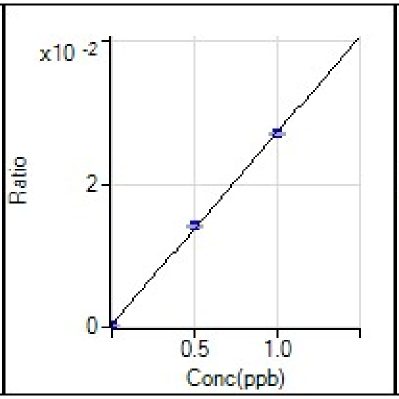

139 La [He]
ISTD: 175 Lu
y = 2.684E-2 x + 3.807E-4
R 0.9998
DL 0.004323
BEC 0.01418

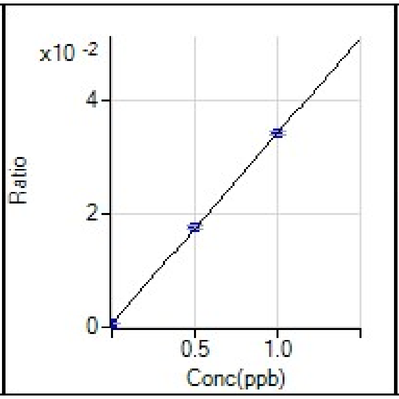

140 Ce [He]
ISTD: 175 Lu
y = 3.365E-2 x + 6.236E-4
R 1.0000
DL 0.005713
BEC 0.01853

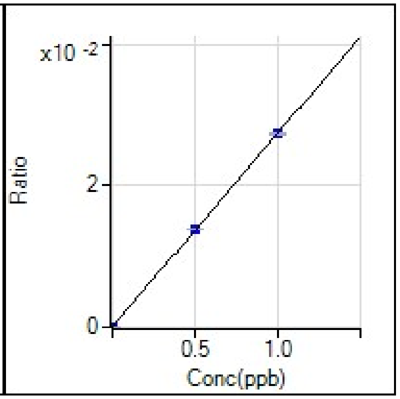

141 Pr [He]
ISTD: 175 Lu
y = 2.730E-2 x + 4.791E-5
R 1.0000
DL 0.001528
BEC 0.001755

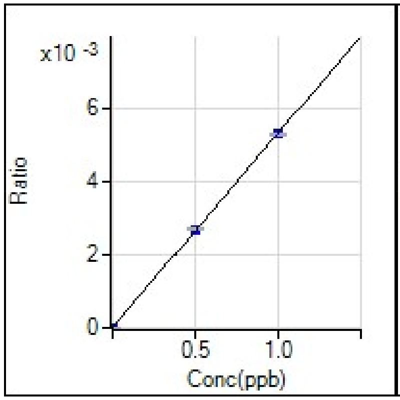

146 Nd [He]
ISTD: 175 Lu
y = 5.330E-3 x + 1.135E-5
R 0.9999
DL 0.002815
BEC 0.002129

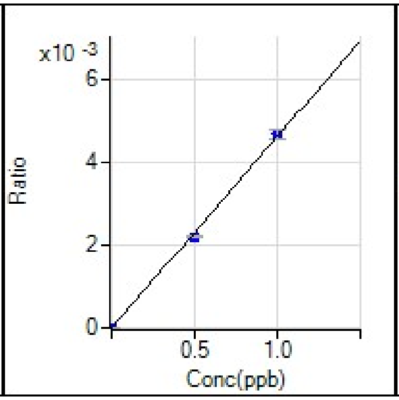

147 Sm [He]
ISTD: 175 Lu
y = 4.635E-3 x + 1.093E-6
R 0.9993
DL 0.001139
BEC 0.0002357

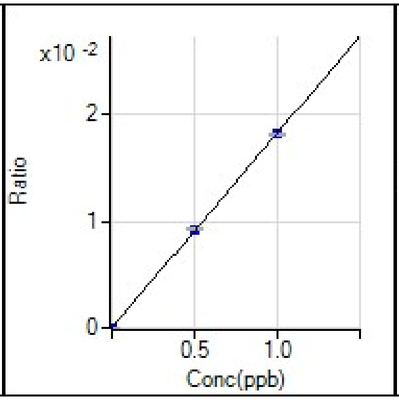

153 Eu [He]
ISTD: 175 Lu
y = 1.823E-2 x + 5.865E-6
R 0.9999
DL 0.0007103
BEC 0.0003217

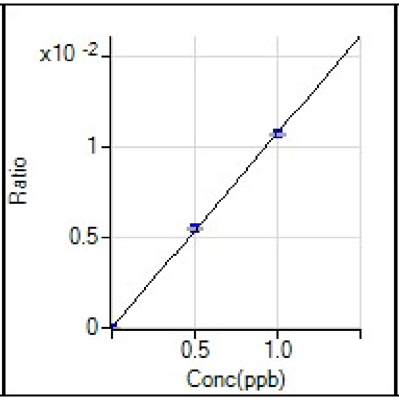

157 Gd [He]
ISTD: 175 Lu
y = 1.073E-2 x + 4.777E-6
R 0.9999
DL 0.001089
BEC 0.0004452

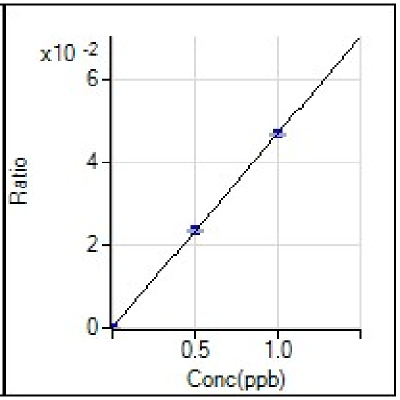

159 Tb [He]
ISTD: 175 Lu
y = 4.704E-2 x + 1.973E-5
R 1.0000
DL 0.0003759
BEC 0.0004194

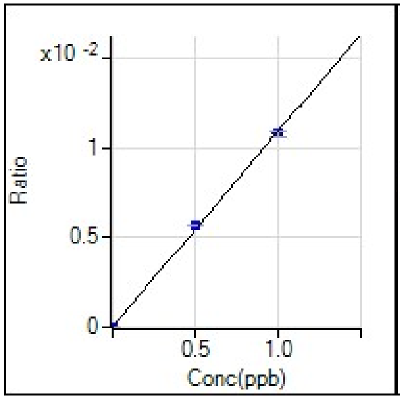

163 Dy [He]
ISTD: 175 Lu
y = 1.092E-2 x + 9.583E-6
R 0.9995
DL 0.00241
BEC 0.0008779

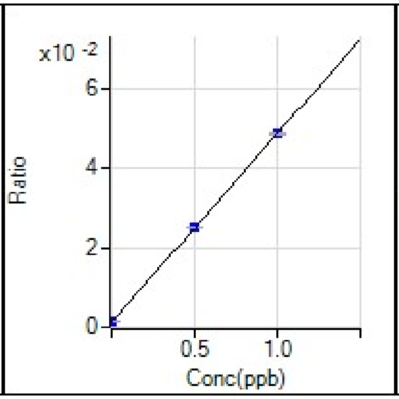

165 Ho [He]
ISTD: 175 Lu
y = 4.745E-2 x + 1.257E-3
R 1.0000
DL 0.002993
BEC 0.02648

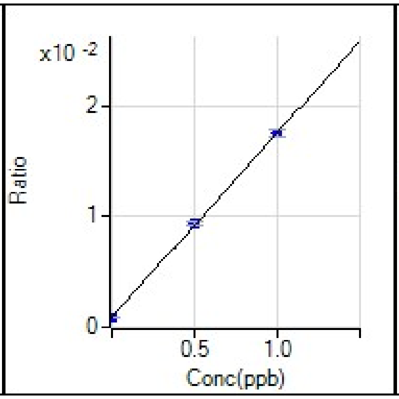

166 Er [He]
ISTD: 175 Lu
y = 1.662E-2 x + 9.173E-4
R 0.9999
DL 0.01185
BEC 0.05521

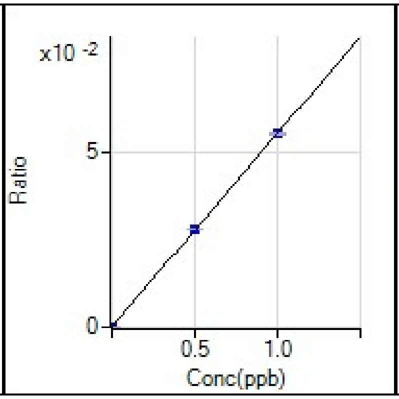

169 Tm [He]
ISTD: 175 Lu
y = 5.522E-2 x + 1.327E-4
R 1.0000
DL 0.001329
BEC 0.002403

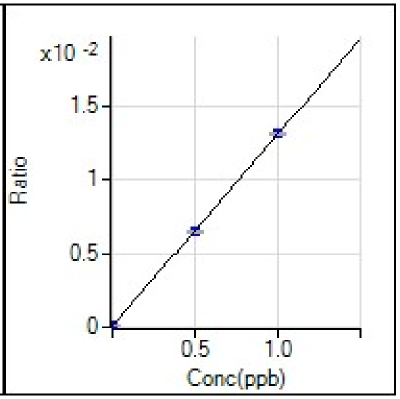

172 Yb [He]
ISTD: 175 Lu
y = 1.296E-2 x + 7.433E-5
R 0.9999
DL 0.003184
BEC 0.005736

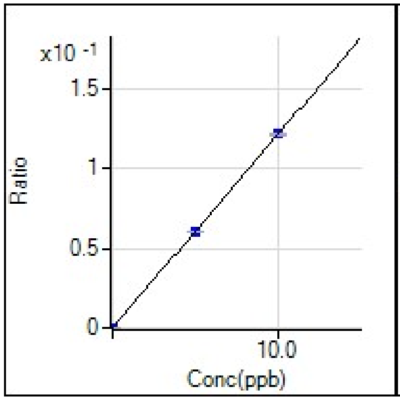

178 Hf [He]
ISTD: 175 Lu
y = 1.210E-2 x + 1.009E-4
R 1.0000
DL 0.0059
BEC 0.008339

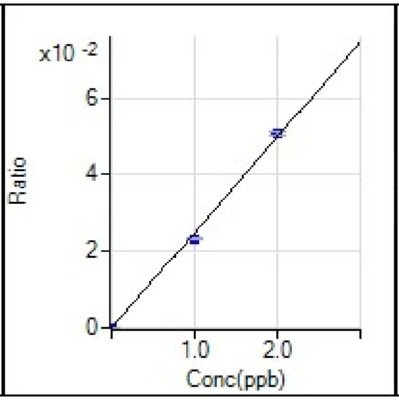

195 Pt [He]
ISTD: 175 Lu
y = 2.491E-2 x + 1.387E-5
R 0.9990
DL 0.0005732
BEC 0.0005566

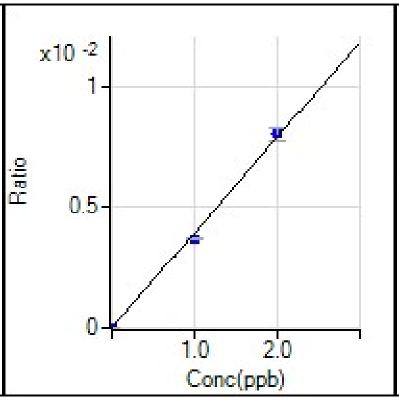

201 Hg [He]
ISTD: 175 Lu
y = 3.941E-3 x + 7.180E-6
R 0.9988
DL 0.0016
BEC 0.001822

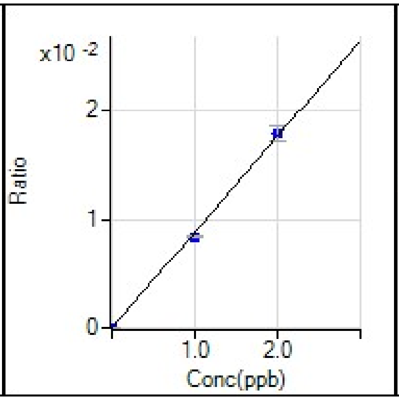

202 Hg [He]
ISTD: 175 Lu
y = 8.835E-3 x + 1.722E-5
R 0.9994
DL 0.001618
BEC 0.001949

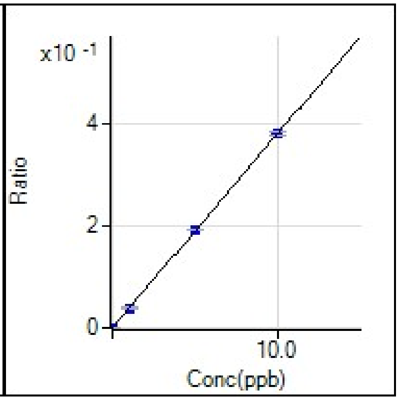

205 Tl [He]
ISTD: 175 Lu
y = 3.795E-2 x + 8.188E-5
R 1.0000
DL 0.001475
BEC 0.002158

Calibration for D:\ICPMH\1\DATA\mozg_24-05-2021.b\001CALB.d

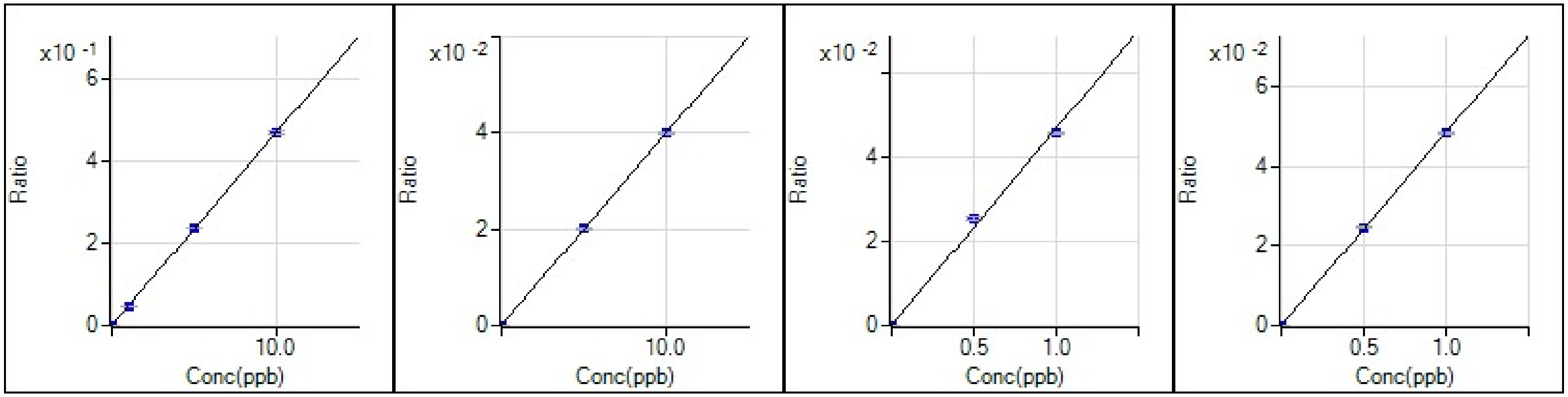

208 Pb [He]	209 Bi [He]	232 Th [He]	238 U [He]
ISTD: 175 Lu	ISTD: 175 Lu	ISTD: 175 Lu	ISTD: 175 Lu
y = 4.682E-2 x + 4.150E-4	y = 4.002E-3 x + 7.644E-5	y = 4.688E-2 x + 5.600E-5	y = 4.844E-2 x + 1.829E-5
R 1.0000	R 1.0000	R 0.9979	R 0.9999
DL 0.006119	DL 0.01216	DL 0.0007835	DL 0.0007294
BEC 0.008865	BEC 0.0191	BEC 0.001194	BEC 0.0003776

Figure S1. The validation protocol of the ICP-MS method.
